# Single-Nucleotide Polymorphisms in the Beta-Tubulin Gene and Its Relationship with Treatment Response to Albendazole in Human Soil-Transmitted Helminths in Southern Mozambique

**DOI:** 10.4269/ajtmh.21-0948

**Published:** 2022-07-18

**Authors:** Berta Grau-Pujol, Javier Gandasegui, Valdemiro Escola, Helena Marti-Soler, Maria Cambra-Pellejà, Maria Demontis, Eric A. T. Brienen, Jose Carlos Jamine, Osvaldo Muchisse, Anelsio Cossa, Charfudin Sacoor, Jorge Cano, Lisette Van Lieshout, Maria Martinez-Valladares, Jose Muñoz

**Affiliations:** ^1^ Barcelona Institute for Global Health (ISGlobal), Hospital Clínic – University of Barcelona, Barcelona, Spain;; ^2^ Centro de Investigação em Saúde de Manhiça (CISM), Maputo, Mozambique;; ^3^ Mundo Sano Foundation, Buenos Aires, Argentina;; ^4^ Instituto de Ganadería de Montaña (CSIC-Universidad de León), Grulleros, León, Spain;; ^5^ Departamento de Sanidad Animal, Facultad de Veterinaria, Universidad de León, Campus de Vegazana, León, Spain;; ^6^ Department of Parasitology, Centre of Infectious Diseases, Leiden University Medical Center (LUMC), Leiden, The Netherlands;; ^7^ Expanded Special Project for Elimination of NTDs, World Health Organization Regional Office for Africa, Brazzaville, The Republic of the Congo

## Abstract

Soil-transmitted helminth (STH) cornerstone control strategy is mass drug administration (MDA) with benzimidazoles. However, MDA might contribute to selection pressure for anthelmintic resistance, as occurred in livestock. The aim of this study is to evaluate the treatment response to albendazole and the relationship with the presence of putative benzimidazole resistance single-nucleotide polymorphisms (SNPs) in the β-tubulin gene of STH in Southern Mozambique. After screening 819 participants, we conducted a cohort study with 184 participants infected with STH in Manhiça district, Southern Mozambique. A pretreatment and a posttreatment stool samples were collected and the STH infection was identified by duplicate Kato-Katz and quantitative polymerase chain reaction (qPCR). Cure rate and egg reduction rates were calculated. Putative benzimidazole resistance SNPs (F167Y, F200T, and E198A) in *Trichuris trichiura* and *Necator americanus* were assessed by pyrosequencing. Cure rates by duplicate Kato-Katz and by qPCR were 95.8% and 93.6% for *Ascaris lumbricoides*, 28% and 7.8% for *T. trichiura*, and 88.9% and 56.7% for *N. americanus*. Egg reduction rate by duplicate Kato-Katz was 85.4% for *A. lumbricoides*, 34.9% for *T. trichiura*, and 40.5% for *N. americanus*. Putative benzimidazole resistance SNPs in the β-tubulin gene were detected in *T. trichiura *(23%) and *N. americanus* (21%) infected participants at pretreatment. No statistical difference was observed between pretreatment and posttreatment frequencies for none of the SNPs. Although treatment response to albendazole was low, particularly in *T. trichiura*, the putative benzimidazole resistance SNPs were not higher after treatment in the population studied. New insights are needed for a better understanding and monitoring of human anthelmintic resistance.

## INTRODUCTION

Approximately 1.5 billion people have a soil-transmitted helminth (STH) infection worldwide. The main STH species are *Ascaris lumbricoides*, *Trichuris trichiura*, and hookworms (*Necator americanus* and *Ancylostoma duodenale*). They are transmitted through the fecal-oral route or skin penetration causing nutritional and physical impairment.^1^

Soil-transmitted helminth control is based on preventive chemotherapy. The WHO recommends mass drug administration (MDA) with benzimidazoles (albendazole or mebendazole) to people at-risk: once a year when prevalence is < 20% and twice a year when < 50%.^1^ After MDA, infective STH stages in the environment can still cause reinfection. But this is not the only MDA destabilizing, periodical MDA could contribute to selection pressure for resistance emergence.^2^^,^^3^

In the last decades, veterinary scientists reported anthelmintic resistance in nematodes infecting livestock, mainly ruminants, after frequent benzimidazoles use.^4^ Benzimidazoles bind to nematode tubulin dimers and inhibit the microtubules formation and stability. In nematodes infecting ruminants, benzimidazole resistance is characterized by single-nucleotide polymorphisms (SNPs) in the β-tubulin encoding gene that reduces binding affinity, specifically, amino acid substitutions from phenylalanine to tyrosine at codons 167 or 200 (F167Y or F200Y), and substitutions from glutamate to alanine or leucine at codon 198 (E198A, E198L).^2^^,^^5^^–^^8^

In human STHs, the decreasing treatment response to benzimidazole is raising concerns about resistance.^9^^,^^10^ In many aspects, STH control in humans is comparable to their control in livestock: large-scale and frequent preventive chemotherapy for a long time, high risk of underdosing (single dose), and lack of resistance monitoring.^11^^,^^12^ However, the relationship between β-tubulin SNPs and benzimidazole response in human STH is still unclear.

Mozambique is a country in sub-Saharan Africa with 53% national STH prevalence before MDA implementation.^13^ Since 2011 until nowadays, MDA has been conducted once or twice a year in schoolchildren, following WHO guidelines.^14^^,^^15^

Considering MDAs conducted in Mozambique, we performed a cohort study to evaluate the treatment response to albendazole of each STH in Manhiça district, Southern Mozambique. In addition, we explored the presence of putative benzimidazole resistance SNPs in the β-tubulin gene by using pyrosequencing and the potential association with treatment response.

## MATERIALS AND METHODS

### Study area and population.

The study was conducted in Manhiça district, Southern Mozambique, a rural setting with a subtropical climate. The main occupations are farming, petty trading, and sugar cane estate employment. The demographic trends in Manhiça district are described in detail elsewhere.^16^

For screening, a regular sampling design was used to obtain a spatial representation of Manhiça district.^17^ Briefly, we divided the whole study area using a regular grid formed by 440 square areas of 1,750 m by 1,750 m resolution. One household was selected by defined area, the closest to the centroid, and then one person between 5 and 15 years of age and one person more than 15 years old were randomly selected per sampling point (Supplemental Figure 1). Further sampling details have been previously described.^18^

Selected individuals who had taken anthelmintics any time during the previous 30 days were excluded.

### Sample size justification.

This study was developed as a nested study of another study that had its main objective was to predict the STH infection prevalence in the study area.^18^ In the absence of updated data on STH prevalence in the study area, we assumed a conservative 50% prevalence in both age groups to ensure a sample size enough to achieve a precision of 5% in the estimation of the 95% confidence intervals (Cis) regardless of the true prevalence. Thus, the estimated sample size required for screening was 384 for each of the age groups (*N* = 768). Considering a 10% loss to follow-up, the final sample size calculation resulted in 423 participants between 5 and 15 years old and 423 participants more than 15 years old (*N* = 846).^18^ The evaluation of treatment response to albendazole in this cohort was a secondary objective and did not have a specific sample size calculation.

### Sample collection.

At screening, two stool samples were collected per participant on two consecutive days. A field worker visited the candidates’ households, invited them to participate and provided written informed consent and study questionnaire. Samples were collected on the two following days after recruitment (Supplemental Figure 1). Collected samples were transported in a cold box to CISM (Centro de Investigação em Saúde de Manhiça) for the laboratory analysis.

Those participants with at least one STH infection detected by microscopy at CISM were requested a third pretreatment stool sample for pyrosequencing analysis, which was collected the day they received treatment with albendazole (400 mg). Moreover, a stool sample 21 days after treatment was collected to assess albendazole treatment response and to conduct pyrosequencing (Supplemental Figure 1).

### Laboratory methods.

#### Microscopy detection.

**Figure 1.**
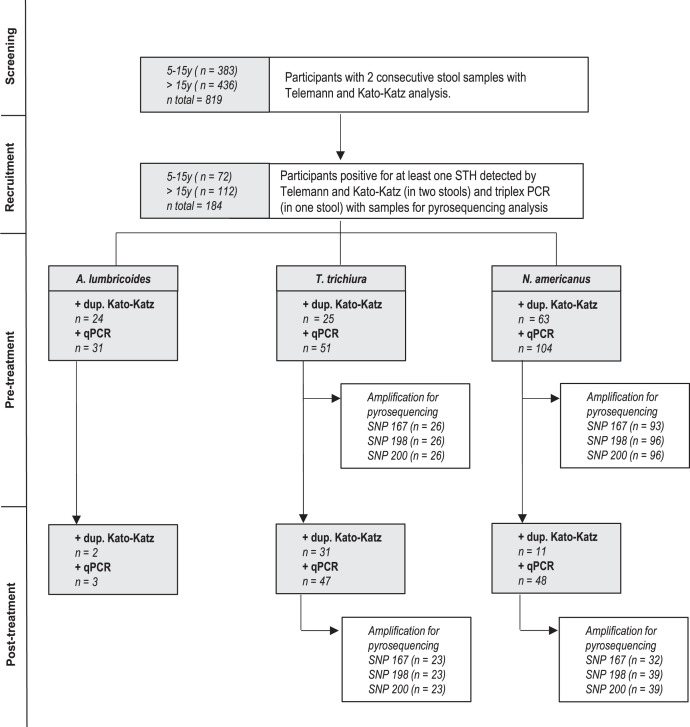
Diagram flow of participants screened, recruited, and followed-up. The number of positive (+) participants per technique at pretreatment and posttreatment is presented, as well as participants’ samples that amplified for pyrosequencing analysis to detect putative benzimidazole resistance single-nucleotide polymorphisms (SNPs).

At screening, two stool samples were evaluated for the identification of STH eggs by microscopy: Telemann concentration technique^19^ and duplicate Kato-Katz thick smear test from Sterlitech Corporation. The procedure and quality control methodology has been described elsewhere^20^ (Figure 1, Supplemental Figure 1).

Posttreatment samples were analyzed by duplicate Kato-Katz with the same procedure above (Figure 1, Supplemental Figure 1).

After microscopy examination, samples were aliquoted and stored at −80°C at CISM for forthcoming molecular analysis.

#### Egg concentration for pyrosequencing.

Soil-transmitted helminth eggs from pretreatment and posttreatment samples were concentrated for later pyrosequencing analysis following the protocol described by Gandasegui et al.^21^ (Supplemental Figure 1). Briefly, the fresh stool was homogenized after shaking with water and glass beads in a 50-mL bottle for 1–2 minutes. Then, the homogenized sample was forced through three metallic sieves of decreasing pore size (150, 80, and 20 µm) using high-pressure tap water. The sediment retained in the 20-µm sieve contained the STH eggs. All sediments retained after egg concentration were stored at −80°C until DNA extraction.

#### DNA extraction.

Unprocessed stool and pretreatment and posttreatment concentrated egg samples were transported under frozen conditions to the University of Leon, Spain. The DNA was extracted from both types of samples using the QIAamp Power Fecal Pro DNA Kit (QIAGEN, Hilden, Germany), following the manufacturer’s instructions. The DNA samples were stored at −20°C until use.

#### Multiplex real-time quantitative polymerase chain reaction to quantify STH infection.

The extracted DNA samples from unprocessed stool samples were stored at −20°C and sent to the Leiden University Medical Center, the Netherlands, for the detection and quantification of parasite-specific DNA by two well-established multiplex real-time PCR detection panels. Panel 1 targets *A. duodenale*, *N. americanus*, *A. lumbricoides*, and *S. stercoralis*, whereas panel 2 targets *T. trichiura* and *Schistosoma* spp. The sequences of primers and probes and the setup of the PCR were based on the published information.^22^^–^^24^ Amplification, detection, and analysis were performed using the CFX real-time detection system (Bio-Rad Laboratories). Negative and positive control samples were included in each PCR run. Cycle threshold (Ct) value results were analyzed using Bio-Rad CFX software (Manager V3.1.1517·0823). The outcome of *Schistosoma* spp. and *S. stercoralis* PCR will not be presented here (Figure 1, Supplemental Figure 1).

#### Triplex-PCR to select stool samples.

Apart from CISM microscopy analysis, all pretreatment and posttreatment samples were also analyzed by triplex conventional PCR at the University of Leon to simultaneously detect *T. trichiura*, *N. americanus*, or *Ascaris lumbricoides* infections undetected by microscopy. Those samples positive for at least on STH detected by microscopy and/or Triplex-PCR were later analyzed by pyrosequencing. We followed the methodology previously described^25^ (Figure 1, Supplemental Figure 1).

#### Pyrosequencing assay.

The pyrosequencing assay to determine the frequency of the resistance alleles related to benzimidazole resistance for each species (*T. trichiura* and *N. americanus*) was performed following the protocol previously described by Gandasegui et al.^21^ Briefly, a first PCR was conducted at the University of León to amplify a fragment of the beta-tubulin gene including the three resistance-associated codons (167, 198, and 200), for each species, in all pre- and posttreatment samples. In all PCRs, plasmid constructions for each species including the susceptible genotype for the three SNPs, the resistant genotype, and a mixture of both as an intermediate genotype (1:1) were included as internal controls for the pyrosequencing assay. The amplified products were then sent to the Instituto Aragonés de Ciencias de la Salud (IACS) where the pyrosequencing technique was performed following the protocol by Gandasegui et al.^21^

### Data analysis.

All statistical analysis was carried out in R Statistical Software Version 3.5.3^26^ (The R Foundation for Statistical Computing, Vienna, Austria) and STATA version 16 (StataCorp., College Station, TX).

Participant population was described using absolute and relative frequency. Socioeconomical status data classification was previously described elsewhere.^18^

#### Treatment response.

All participants who are STH positive by any of the techniques (Telemann, Kato-Katz, or triplex PCR) in any of the two stool samples collected at screening were recruited and were assessed for treatment response.

Treatment response was evaluated considering the first stool sample collected during screening (pretreatment sample) and a follow-up sample collected 21 days after treatment with albendazole (posttreatment sample) (Supplemental Figure 1).

Participants’ eggs per gram (EPG) of feces at pretreatment and posttreatment detected by duplicate Kato-Katz were expressed using geometric mean and 95% CIs. For Kato-Katz, we used WHO thresholds^27^ to classify the intensity of infection per STH. Quantitative polymerase chain reaction (qPCR) Ct-values were expressed using median and upper and lower quartiles.

The cure rate (CR) for duplicate Kato-Katz and for qPCR were calculated per STH. The CR is defined by the percentage of subjects who after treatment were negative by the diagnostic technique. Kato-Katz CR was calculated as (1 − [number of subjects excreting eggs at posttreatment/number of subjects excreting eggs at pretreatment])*100. Quantitative polymerase chain reaction CR was calculated as (1 − [number of subjects with detectable DNA at posttreatment/number of subjects with detectable DNA at pretreatment])*100. Cure rates between age groups were compared with Fisher’s exact test.

The egg reduction rate (ERR) for duplicate Kato-Katz was also determined and calculated as (1 − geometric mean egg counts at posttreatment/geometric mean egg counts at pretreatment)*100. Standard errors of ERR were obtained with bootstrap analysis using R package “boot.” Egg reduction rates between age groups were compared with the Wilcoxon rank sum test.

#### Presence and frequencies of putative benzimidazole resistance SNPs.

We described the absolute and relative frequency of participants that presented eggs with putative benzimidazole resistance SNPs at positions 167, 198, and 200 of the β-tubulin gene for *T. trichiura* and the isotype-1 beta-tubulin for *N. americanus* at pretreatment and posttreatment. We compared pretreatment SNPs frequencies with posttreatment frequencies in each STH using Fisher’s exact test.

We calculated the paired difference between EPG at pretreatment and at posttreatment, the paired difference between Ct value at pretreatment and at posttreatment, and the paired difference between SNP frequency at pretreatment and posttreatment in *N. americanus.* We measure Spearman’s rank correlation between EPG and SNP frequency differences and Ct value and SNP frequencies differences. The small number of infected participants with SNPs hamper this analysis for *T. trichiura.*

### Ethics.

The study followed the Declaration of Helsinki (version of Fortaleza, Brazil, October 2013), current ICH-GCP guidelines, and all applicable national and local regulatory requirements (Spanish Royal Decree 1090/2015). National Bioethics Committee for Health in Mozambique granted approval for this study (Ref.:517/CNBS/17). Participation in this study was voluntary. We obtained written informed consent in either Portuguese or Changana from all study participants older than 18 years all. Caregivers provided written informed consent for participants under 18 years all. Participants between 15 and 17 years old also provided written informed assent. For illiterate caregivers, informed consent was conducted in presence of a study independent literate witness.

## RESULTS

### Participants description.

In total, 819 participants were screened, of which 22.5% (*N* = 184) were positive for at least one STH either by Telemann from two stools, Kato-Katz from two stools, or Triplex PCR from one stool: 4.5% for *A. lumbricoides*, 6.5% for *T. trichiura*, and 14.5% for *N. americanus*. All positive participants were followed up to evaluate treatment response and to detect putative benzimidazole resistance SNPs (Figure 1).

The proportion of participants included in the cohort that was between 5 and 15 years old was 39.1%. Female participants were 56.5% and 64.7% of participants lived in a low socioeconomic household (Table 1).

**Table 1:** Description of study participants’ characteristics by frequency and percentage, *n* (%)

	Participants 5–15 years old	Participants > 15 years old	All participants
Gender			
Female	31 (43.1)	73 (65.2)	104 (56.5)
Male	41 (56.9)	39 (34.8)	80 (43.5)
Age (years)			
5–10	43 (59.7)	–	43 (23.4)
10–15	29 (40.3)	–	29 (15.8)
15–34	–	33 (29.5)	33 (17.9)
35–54	–	29 (25.9)	29 (15.8)
55–74	–	38 (33.9)	38 (20.7)
≥ 75	–	12 (10.7)	12 (6.5)
Household socioeconomical status			
Rich	5 (6.9)	9 (8.0)	14 (7.6)
Middle class	18 (25.0)	33 (29.5)	51 (27.7)
Poor	49 (68.1)	70 (62.5)	119 (64.7)

### Treatment response evaluation.

At baseline, 33.3% of *A. lumbricoides* infections were classified as having moderate intensity and 66.6% were classified as low intensity. All *T. trichiura* infections were low intensity. Finally, 4.6% of *N. americanus* infections were moderate intensity and 95.4% were low.

After treatment, 2/24 participants were positive for *A. lumbricoides* by duplicate Kato-Katz and 3/31 participants by qPCR. Regarding *T. trichiura*, 31/25 participants were positive by duplicate Kato-Katz and 47/51 participants were positive by qPCR. Concerning *N. americanus,* 11/63 participants were positive by duplicate Kato-Katz and 48/104 participants by qPCR. All *T. trichiura* and *N. americanus* infections were classified as low intensity and only one participant infected with *A. lumbricoides* was classified as high intensity of infection (Table 2).

**Table 2:** Number of participants positive for STH infection, intensity of infection and cure rate, and egg reduction rate per Kato-Katz and qPCR per each group and total study population

	5–15 years old (*N* = 72)			> 15 years old (*N* = 112)			All (*N* = 184)		
	*A. lumbricoides*	*T. trichiura*	*N. americanus*	*A. lumbricoides*	*T. trichiura*	*N. americanus*	*A. lumbricoides*	*T. trichiura*	*N. americanus*
Number of participants positive for infection									
Pretreatment duplicate Kato-Katz (%)	13 (18.1)	14 (19.4)	22 (30.6)	11 (9.8)	11 (9.8)	41 (36.6)	24 (13.0)	25 (13.6)	63 (34.2)
Pretreatment qPCR (%)	14 (19.4)	29 (40.3)	33 (45.8)	17 (9.2)	22 (19.6)	71 (63.4)	31 (16.8)	51 (27.7)	104 (56.5)
Posttreatment duplicate Kato-Katz (%)	2 (2.8)	18 (25.0)	7 (9.7)	0 (0.0)	13 (11.6)	3 (2.7)	2 (1.1)	31 (16.8)	11 (6.0)
Posttreatment qPCR (%)	2 (2.8)	29 (40.3)	21 (29.2)	1 (0.9)	18 (16.1)	27 (24.1)	3 (1.6)	47 (25.5)	48 (26.1)
Intensity of infection									
Pretreatment duplicate Kato-Katz geometric mean EPG (95% CI)	2,052 (829–5077)	122 (47–315)	164 (84–320)	759 (238–2,413)	32 (18–58)	125 (75–211)	1,366 (687–2714)	66 (38–116)	132 (90–194)
Pretreatment qPCR median Ct value (Q1–Q3)	25 (22–27)	28 (26–29)	28 (26–29)	25 (21–27)	30 (29–32)	28 (25–30)	25 (21–27)	29 (26–31)	28 (25–30)
Posttreatment duplicate Kato-Katz geometric mean EPG (95% CI)	617 (0–0)	66 (36–122)	84 (24–300)	0 (0–0)	29 (17–51)	134 (46–394)	358 (1–184,879)	43 (29–66)	92 (44–194)
Posttreatment qPCR median Ct value (Q1–Q3)	28 (23–33)	28 (27–29)	74 (22–247)	25 (25–25)	29 (27–32)	29 (27–32)	25 (23–33)	28 (27–31)	29 (27–32)
CR									
Kato-Katz CR, %	84.6	−28.6	68.2*	100.0	−18.2	92.7*	91.7	−24.0	82.5
qPCR CR, %	85.7	0.9*	36.4*	94.1	18.2*	62.0*	90.3	7.8	53.8
ERR									
Kato-Katz geometric mean ERR, %	76.0	49.2†	53.9	100.0	10.1†	−13.3	85.4	34.9	30.5

CR = cure rate; Ct = cycle threshold; EPG = eggs per gram; ERR = egg reduction rate; qPCR = quantitative polymerase chain reaction; STH = soil-transmitted helminth.

* Fisher’s exact test, *P* value < 0.05 comparing 5–15 years old and < 15 years old participants.

† Wilcoxon rank sum test, *P* value < 0.05 comparing 5–15 years old and < 15 years old participants.

Among participants infected with hookworm, only *N. americanus* was detected by PCR.

**Figure 2.**
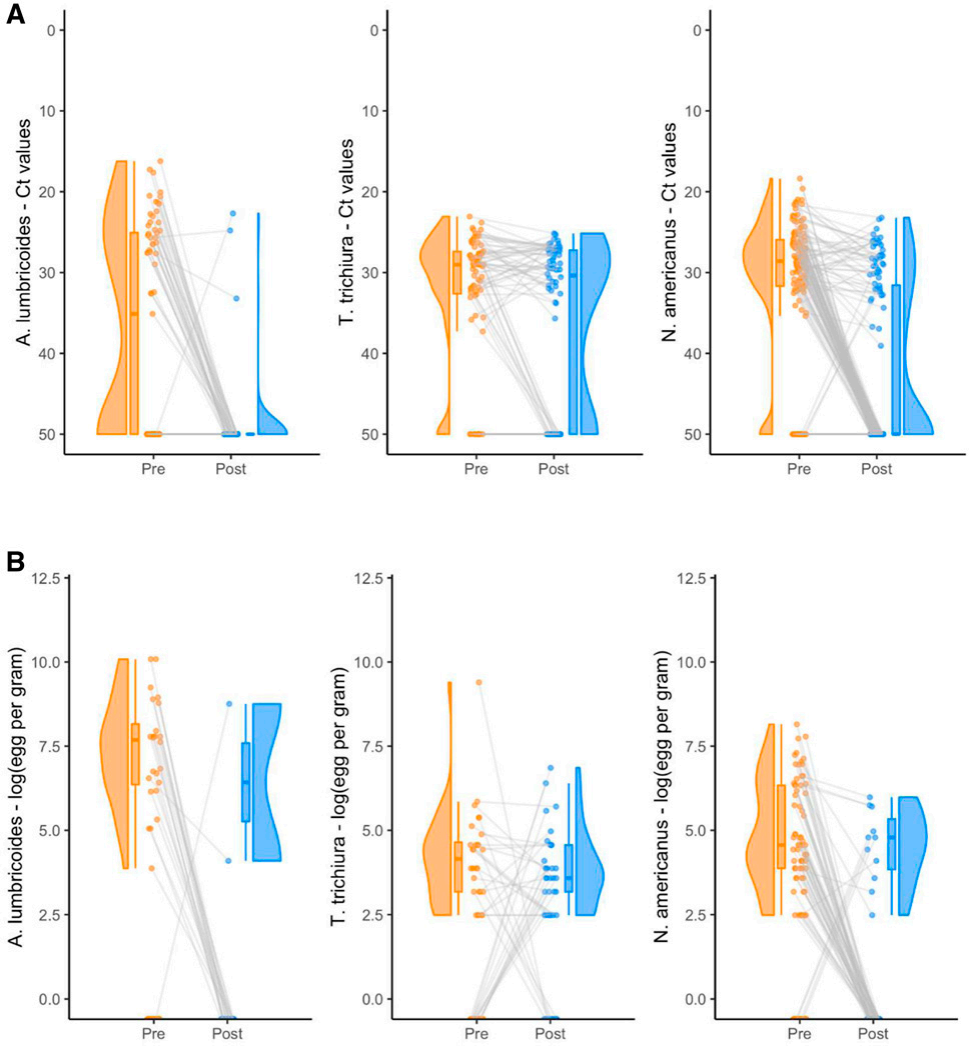
(**A**) Logarithm of eggs per gram (EPG) at pretreatment and posttreatment per soil-transmitted helminth (STH) species. (**B**) Cycle threshold (Ct) values at pretreatment and posttreatment per STH species. This figure appears in color at www.ajtmh.org.

Soil-transmitted helminth infection in some participants was not detected at pretreatment but it was detected posttreatment. *Ascaris lumbricoides* infection of one participant was only detected at posttreatment by duplicate Kato-Katz and qPCR. *Trichuris trichiura* infection of 13 participants was not detected at pretreatment by duplicate Kato-Katz but at posttreatment. *Necator americanus* infection of three participants was only detected at posttreatment by duplicate Kato-Katz and qPCR (Figure 2).

Cure rate detected by duplicate Kato-Katz for *A. lumbricoides* was 91.7% (84.6% in 5–15 years old and 100% in > 15 years old, *P* value = 0.494), for *T. trichiura* was −24% (−28.6% in 5–15 years old and −18.2% in > 15 years old, incalculable Fisher’s exact test), and for *N. americanus* 82.5% (68.2% in 5–15 years old and 92.7% in > 15 years old, *P* value = 0.016). Cure rate detected by qPCR for *A. lumbricoides* was 90.3% (85.7% in 5–15 years old and 94.1% in > 15 years old, *P* value = 0.425), for *T. trichiura* was 7.8% (0.9% in 5–15 years old and 18.2% in > 15 years old, *P* value < 0.001), and for *N. americanus* was 53.8% (36.4% in 5–15 years old and 62.0% in > 15 years old, *P* value = 0.029, *P* value = 0.013) (Table 2).

Egg reduction rate obtained by duplicate Kato-Katz for *A. lumbricoides* was 76% (85.4% in 5–15 years old and 100% in > 15 years old, *P* value > 0.05), for *T. trichiura* was 34.9% (49.2% in 5–15 years old and 10.1% in > 15 years old, *P* value = 0.04), and for *N. americanus* was 30.5% (53.9% in 5–15 years old and −13.3% in > 15 years old, *P* value > 0.05) (Table 2) (Supplemental Table 2).

### Presence of putative benzimidazole resistance SNPs in *T. trichiura* and *N. americanus.*

**Figure 3.**
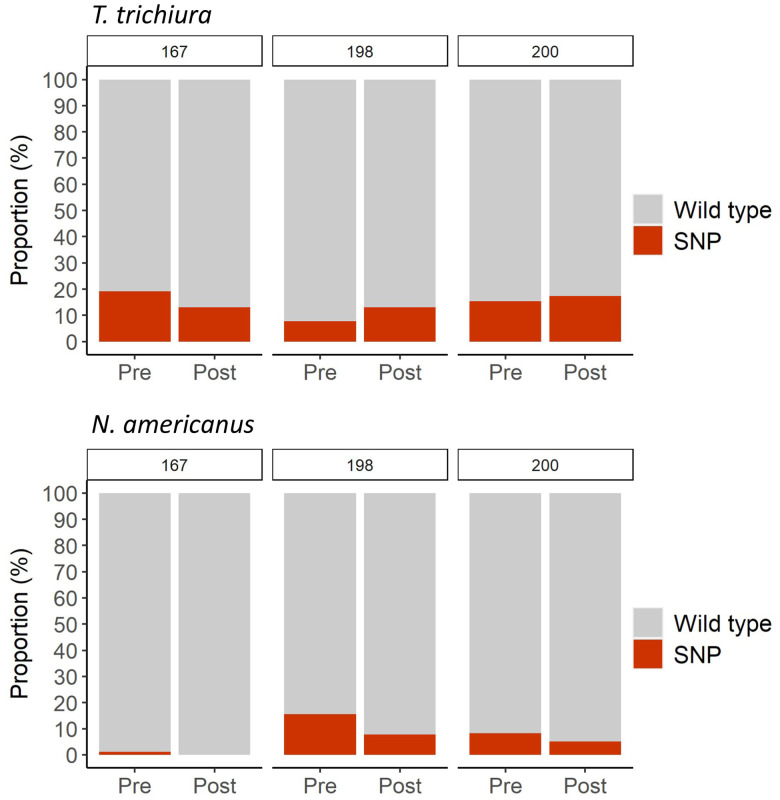
Proportion (%) of participants who presented eggs wild type (Wt) or with single-nucleotide polymorphism (SNP) at positions 167, 198, and 200 for *Trichuris trichiura* and *Necator americanus* infection at pretreatment and posttreatment. This figure appears in color at www.ajtmh.org.

We detected 6/26 participants infected by *T. trichiura* with any of the three putative benzimidazole resistance SNPs at pretreatment: five participants showed the SNP at codon 167, two at codon 198, and 4 at codon 200; in addition, one participant had an infection with the three SNPs and three participants had an infection with SNPs 167 and 198. At posttreatment, we detected 4/26 participants infected by *T. trichiura* with at least one SNP: three of them at codon 167, three at codon 198, and four at codon 200; moreover, two participants had an infection with the three SNPs, and one had an infection with SNPs 167 and 200 (Supplemental Table 1). No statistical difference was observed between the overall frequencies at pretreatment and at posttreatment of each SNP (Figure 3).

Regarding *N. americanus*, we detected 20/93 participants with an infection carrying at least one SNP at pretreatment: 1 participant with an infection with SNP 167, 15 participants with an infection with SNP 198, and 8 with SNP 200; in addition, 4 participants had an infection with a SNP at codon 198 and 200. At posttreatment, we detected 5/32 participants infected by *N. americanus* with at least one SNP: no participants with an infection with SNP 167, 3/39 with an infection with SNP 198, and 2/39 with an infection with SNP 200; none of the participants had a *N. americanus* infection with more than one SNP (Supplemental Table 1). No statistical difference was observed between overall pretreatment and posttreatment frequencies for none of the SNPs (Figure 3).

### Correlation between putative benzimidazole resistance SNPs and treatment response.

One participant presented a *T. trichiura* infection carrying a higher SNP frequency at posttreatment (30.4%) than at pretreatment (16.7%) and displayed a negative ERR (−14.3%).

Regarding *N. americanus*, three participants’ infection presented the same SNP at pretreatment and at posttreatment and only one displayed greater 200 SNP frequencies at posttreatment (24.3%) than at pretreatment (14.2%). The participant’s ERR was not obtained since STH infection was not detected by Kato-Katz at posttreatment.

For *N. americanus*, we did not observe any correlation between the difference in EPG and the difference on SNP frequency and between the difference on Ct-values and the difference on SNP frequency at pretreatment and posttreatment of *N. americanus* for none of the SNPs.

## DISCUSSION

This cohort study exhibited that *T. trichiura* treatment response to albendazole was low in Manhiça district, Southern Mozambique. Few study participants with *T. trichiura* and *N. americanus* infections were carrying the benzimidazole resistance SNPs previously described in nematodes infecting livestock. However, treatment response to albendazole was not observed to be related with SNPs presence in human STHs in the current study. Thus, other mechanisms could be triggering treatment failure.

The aim of our study was to evaluate the efficacy of the deworming drug used in Mozambique—albendazole—against the most prevalent STH, and its possible association with the presence of SNPs at the beta-tubulin gene. We observed −24% *T. trichiura* CR evaluated by Kato-Katz and 7.8% *T. trichiura* CR by qPCR in Manhiça district, which goes in accordance with systematic reviews, meta-analysis on STH drugs’ efficacy^9^^,^^28^^,^^29^ and randomized controlled trials.^30^ In our case, several *T. trichiura* infections were only detected at posttreatment by Kato-Katz, thus, CR by Kato-Katz was negative for this worm. Concerning *N. americanus*, CR by Kato-Katz was 82.5%, higher than in other studies,^9^^,^^28^^–^^30^ and 53.8% by qPCR, lower than a trial in Tanzania^30^ but similar to an efficacy study in Timor-Leste.^31^ In addition, both *N. americanus* CR (calculated by Kato-Katz and qPCR) were lower in 5–15 years old, a more exposed population to anthelmintics during MDA, and thus, higher selection pressure. Regarding *A. lumbricoides*, both CRs (91.7% by Kato-Katz and 90.3% by qPCR) were similar to other studies.^9^^,^^28^^–^^31^ Thus, our findings of a reduced treatment response are comparable to those observed across other different STH endemic regions, which highlights the reduced effectiveness of the main STH control strategy, the MDA with a benzimidazole drug.

For *A. Lumbricoides* and *N. americanus*, CR by qPCR was lower than CR by Kato-Katz. As described elsewhere,^18^ this study was conducted in a low-intensity infection area where qPCR was more sensitive at low-intensity infections, such those at posttreatment, than Kato-Katz.^18^^,^^32^^,^^33^ Thus, we observed a lower qPCR CR than Kato-Katz. However, we observed a negative CR by Kato-Katz for *T. trichiura:* participants positive at posttreatment but with an undetected infection of this STH at baseline. The reason for that could be prepatent infections at baseline hardly detected by Kato-Katz or the day-to-day egg excretion variability.^34^

Geometric mean ERR for *T. trichiura* and for *N. americanus* were 34.9% and 30.5%, respectively, lower than in other studies.^9^^,^^29^^,^^35^ *Ascaris lumbricoides* ERR was much lower than observed before^9^^,^^29^ since one of the two infections at posttreatment was not detected at pretreatment by Kato-Katz.

Treatment failure could be triggered by multiple factors. Poor drug quality and suboptimal dosing could induce low treatment response. Concerning the host, high intensity of infection might interfere with anthelmintic pharmacokinetics and reduce drug bioavailability.^12^^,^^36^ Time since last meal, nutrition status, intestinal transit, and microbiome could also influence on treatment failure.^9^^,^^12^^,^^35^^,^^37^^–^^39^ In our study, valid drugs were stored in hospital pharmacy. But participants were treated following MDA guidelines (albendazole single dose of 400 mg), what could be suboptimal depending on the several factors mentioned above. Regarding the host, most infections were low intensity, favoring drug bioavailability.^36^ However, we could not consider time since last meal, participant’s nutrition status, microbiome, or intestinal transit. Nevertheless, our results are similar to efficacy studies in other endemic areas, which show that ERRs against *N. americanus* and *T. trichiura* decreased over time to below WHO’s reference value.^10^^,^^29^ Hence, anthelmintic resistance could be emerging.

In livestock nematodes, helminths carrying SNPs 167, 198, and 200 in the β-tubulin gene are associated with poor benzimidazole treatment response.^38^^,^^40^ Most studies in STH humans only analyzed the presence of these SNPs, which were sometimes absent,^41^^,^^42^ but most studies were not designed to evaluate their association with treatment response. Only a study with unpaired pretreatment and posttreatment samples observed a higher SNP 200 frequency in *T. trichiura* at posttreatment.^43^

In Manhiça district, SNPs previously described in livestock nematodes were present at pretreatment in *T. trichiura* and *N. americanus* but at a very low frequencies. To consider that the presence of SNPs is related with anthelmintic resistance, their frequency should be higher after treatment, since only those parasites with the resistant alleles would survive anthelmintic treatment.^8^^,^^44^ But, we did not observe a higher SNP frequency at posttreatment than at pretreatment, with the exception of one participant infected by *T. trichiura* and one *N. americanus* infected.

A too simplistic description of anthelmintic resistance could have been assumed for benzimidazoles in STH: a unique candidate gene involved with drug mechanism of action. Other resistance mechanisms might be occurring, multiple genes or alleles contributing synergically. Therefore, genome-wide approaches might unravel further genes involved.^45^

However, the low number of infections where we detected a putative benzimidazole SNP is a limitation. It unabled multifactorial analysis where confounders such as nutrition status could be accounted.^46^ The reason could be the low allele population frequencies but also the limit number of samples analyzed by pyrosequencing.^43^ Pyrosequencing-PCR assay sensitivity is lower than diagnostic-PCR since pyrosequencing-PCR must amplify a fragment of the β-tubulin that is only repeated once in the genome while diagnostic-PCRs are performed on highly repetitive genome sequences.^47^^,^^48^ Second, only one stool sample analysis at posttreatment may be affected by day-to-day egg excretion variation.^49^

Mass drug administration is a safe and inexpensive strategy that relies on continued efficacy of anthelmintics. However, it does not prevent from reinfection and contributes to drug selection pressure for resistant strains.^50^ Lately, current recommended deworming drugs’ efficacy has decreased^9^—anthelmintic resistance could be emerging. To prevent it and control it, policy makers’ decisions should account for drug combination, treatment frequency, refugia, and underdosing.^12^ Moreover, an anthelmintic efficacy surveillance system should be created and highly sensitive diagnostics such as qPCR^34^ and field tools should be used.^51^ Furthermore, human anthelmintic resistance mechanisms and genetic markers should be further described. Ad interim, other nondrug driven STH control strategies, such as water, sanitation and hygiene improvements, should be integrated in national control programs to mitigate anthelmintic resistance emergence.

## Supplemental files


Supplemental materials
