# Supplementary material for: Single-Nucleotide Polymorphisms in the Beta-Tubulin Gene and Its Relationship with Treatment Response to Albendazole in Human Soil-Transmitted Helminths in Southern Mozambique
Source: Am J Trop Med Hyg. 2022 Jul 18;107(3):649–57. doi: 10.4269/ajtmh.21-0948 (PMC9490645; doi:10.4269/ajtmh.21-0948)
Supplement: Supplementary file 1 [file tpmd210948.SD1.pdf]

Supplemental Figure S1. Diagram flow of participants' screened and followed-up.

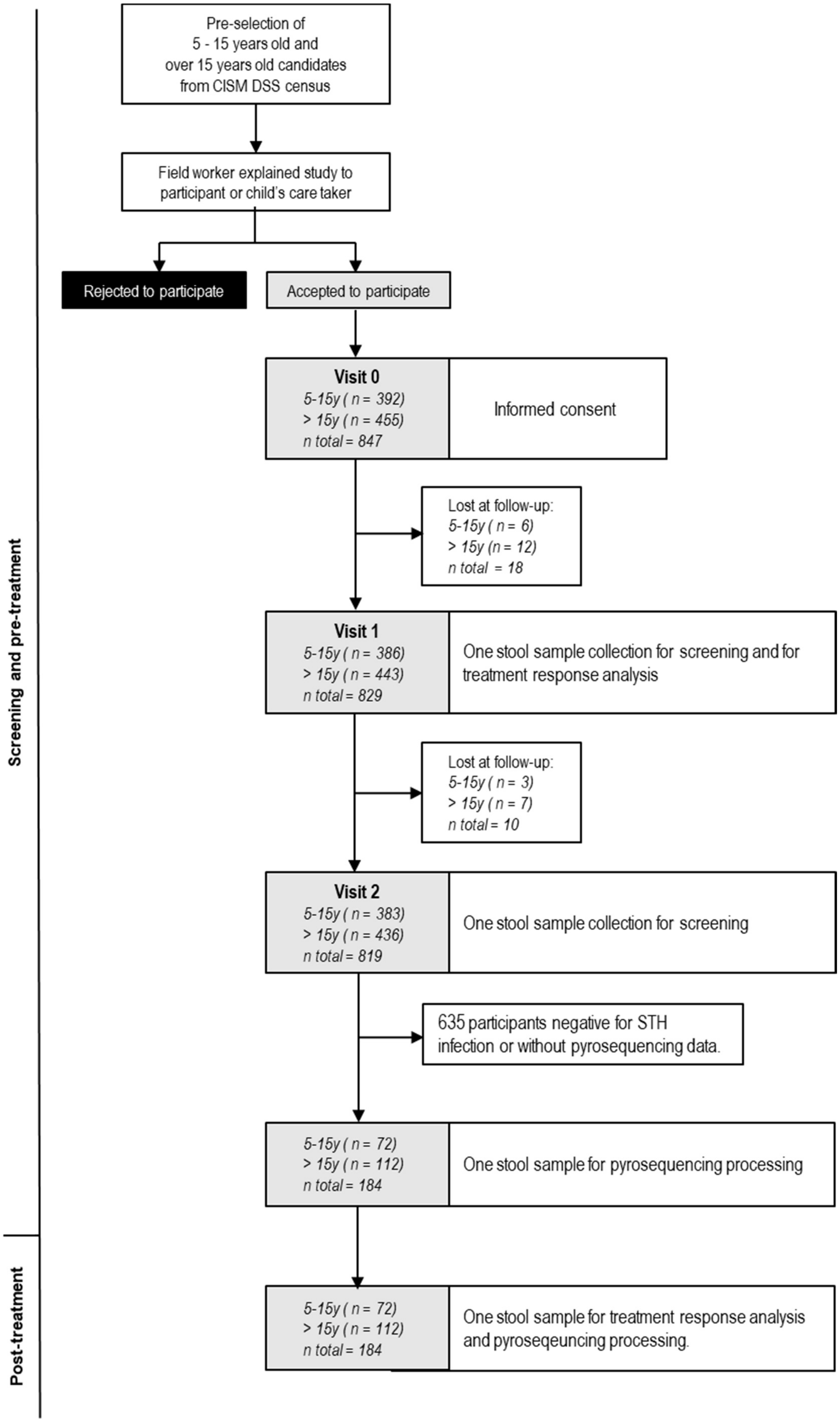

Supplemental Table S1. Single nucleotide polymorphism (SNP) frequencies of participants carrying putative benzimidazole resistance SNPs *T. trichiura* infections (A) *or N. americanus* infections (B) at pre-treatment and at post-treatment.

A

	Pre-treatment			Post-treatment		
	SNP 167 (%)	SNP 198 (%)	SNP 200 (%)	SNP 167 (%)	SNP 198 (%)	SNP 200 (%)
1	11.9		16.7		12.0	30.4
2	14.9	18.8	23.9			
3	12.1		22.5			
4	17.5		16.8			
5	10.1					
6		10.6				
7				22.6	13.1	30.4
8				17.5	11.6	24.9
9				14.6		18.8

B

	Pre-treatment			Post-treatment		
	SNP 167 (%)	SNP 198 (%)	SNP 200 (%)	SNP 167 (%)	SNP 198 (%)	SNP 200 (%)
1	16.7					
2		15.0				
3		11.3				
4		19.2	10.4			
5		14.1	10.2			
6		15.3				
7		17.0				
8		20.5				
9		20.7				
10		15.2				
11		21.4	16.4		16.3	
12		16.0				
13		16.8			15.3	
14		16.6				
15		10.9	14.2			24.3
16		15.6				
17			49.0			
18			17.6			
19			10.4			
20			11.4			
21					15.2	
22						10.9

Supplemental Table S2. Egg reduction rate (ERR) obtained by Kato-Katz per each group and total study population, and its corresponding standard error (SE) calculated by bootstrap analysis.

	A. lumbricoides	*T. trichiura*	*N. americanus*

	ERR	SE	ERR	SE	ERR	SE
**5-15 years old (n = 72)**	76.0	0	49.2	27.9	53.9	12.24
**> 15 years old (n = 112)**	100	0	10.1	39.3	-13.3	162.5
**All (n = 184)**	85.4	0	34.9	23.8	30.5	27.0
